# Type 1 interferons promote *Staphylococcus aureus* nasal colonization by inducing phagocyte apoptosis

**DOI:** 10.1038/s41420-024-02173-2

**Published:** 2024-09-13

**Authors:** Emilio G. Vozza, Alanna M. Kelly, Clíodhna M. Daly, Sinead A. O’Rourke, Simon R. Carlile, Brenda Morris, Aisling Dunne, Rachel M. McLoughlin

**Affiliations:** 1 Host-Pathogen Interactions Group, School of Biochemistry and Immunology, Trinity Biomedical Sciences Institute, Trinity College Dublin, Dublin, Ireland; 2 Molecular Immunology Group, School of Biochemistry and Immunology, Trinity College Dublin, Dublin, Ireland

**Keywords:** Antimicrobial responses, Cell death and immune response, Mucosal immunology, Immune cell death

## Abstract

*Staphylococcus aureus* is an important human commensal which persistently colonizes up to 30% of the human population, predominantly within the nasal cavity. The commensal lifestyle of *S. aureus* is complex, and the mechanisms underpinning colonization are not fully understood. *S. aureus* can induce an immunosuppressive environment in the nasal tissue (NT) by driving IL-10 and IL-27 to facilitate nasal colonization, indicating that *S. aureus* has the capacity to modulate the local immune environment for its commensal habitation. Mounting evidence suggests commensal bacteria drive type 1 interferons (IFN-I) to establish an immunosuppressive environment and whilst *S. aureus* can induce IFN-I during infection, its role in colonization has not yet been examined. Here, we show that *S. aureus* preferentially induces IFN signaling in macrophages. This IFN-I in turn upregulates expression of proapoptotic genes within macrophages culminating in caspase-3 cleavage. Importantly, *S. aureus* was found to drive phagocytic cell apoptosis in the nasal tissue during nasal colonization in an IFN-I dependent manner with colonization significantly reduced under caspase-3 inhibition. Overall, loss of IFN-I signaling significantly diminished *S. aureus* nasal colonization implicating a pivotal role for IFN-I in controlling *S. aureus* persistence during colonization through its ability to induce phagocyte apoptosis. Together, this study reveals a novel strategy utilized by *S. aureus* to circumvent host immunity in the nasal mucosa to facilitate nasal colonization.

## Introduction

Whilst *S. aureus* is an adeptly skilled pathogen, it is also a major human commensal found at many anatomical sites but primarily in the anterior nares, where it colonises up to 30% of healthy adults [1, 2]. However, our understanding of nasal colonization is limited, particularly in the context of how *S. aureus* impacts host immunity to facilitate long-term persistence. *S. aureus* is skilled at manipulating its local environment to maintain its niche during infection where it can induce an anti-inflammatory response to suppress both the innate and adaptive arms of the immune system [3–5]. Recently, it has been shown that *S. aureus* exploits immunosuppressive responses to facilitate persistence during colonization by inducing the production of the cytokines IL-27 and IL-10 from myeloid cells within the nasal cavity [6]. IL-10 acts to suppress local effector T cell responses resulting in a blunted IL-17 and IL-22 response. IL-22 reduces *S. aureus* nasal colonization by promoting antimicrobial peptides (AMPs) and limiting staphylococcal ligands that enable *S. aureus* attachment to the nares [7] while IL-17 similarly limits *S. aureus* nasal colonization by inducing AMPs, but also by promoting the recruitment of neutrophils [8, 9]. Recently, neutrophil recruitment was found to mediate transiency of *S. aureus* skin colonization in a xenograft humanized mouse model highlighting the importance of neutrophils in preventing/controlling *S. aureus* colonization [10]. Consequently, for a colonizing strain to effectively establish its niche, it must have the capacity to overcome the influx of phagocytes, which would otherwise eliminate the bacterium. How *S. aureus* does this in nasal tissue, and whether *S. aureus* directly suppresses phagocytes to establish its persistence at this site, remains to be established.

IFN-I are a group of pleiotropic cytokines including IFN-α and IFN-β as well as several other variants which have been well characterized in response to viral infection [11]. However, a growing body of evidence has highlighted the importance of IFN-I in the immune response to bacteria which can be beneficial/detrimental depending on the bacteria and the circumstance. For instance, in *Listeria monocytogenes* infection, IFN-I sensitize lymphocytes to apoptosis whilst suppressing macrophage activation by IFN-γ [12]. However, in *Brucella abortus* infection, IFN-I is host protective by inducing the expression of guanylate-binding proteins to enhance AIM2-mediated immune cell activation [13]. In *S. aureus* infection, IFN-I can have a differential impact depending on the site of infection and the nature of the infecting strain. In the skin, inhibition of *S. aureus* induced SOCS-1 enhances phagocytosis and nitric oxide production by BMDMs resulting in increased bacterial killing, which was mediated by IFN-I [14]. In contrast, in an *S. aureus* pneumonia model, IFN-I exacerbates disease increasing lung pathology by enhancing proinflammatory cytokines whilst also enhancing the number of DCs and NK cells [15]. However, IFN-I has also been shown to enhance *S. aureus* killing in a pneumonia model, using LAC USA300, by driving granzyme B expression, which enhanced *S. aureus* killing by human neutrophils in an IFN-I dependent manner [16]. Importantly, these two studies differed in the strains used, with Parker et al., showing that strain 502a was a significantly greater inducer of IFN-I then LAC USA300. Thus *S. aureus* has the potential to induce IFN-I in a strain dependent manner suggesting that the impact of IFN production may have differential consequences depending on the anatomical site.

It is currently unknown what impact IFN-I has on *S. aureus* nasal colonization. In Systemic Lupus Erythematosus (SLE), up to 50% of patients exhibit excessively high levels of IFN-I [17, 18]. *S. aureus* colonizes up to 50% of skin lesions in patients and IFN-I decreases barrier gene expression, enhancing *S. aureus* adherence to keratinocytes. As such, this suggests IFN-I may enhance the commensal lifestyle of *S. aureus*. Recently, tonic IFN-I signaling in response to the gut commensal, *Bacteroides fragilis*, was found to drive IL-10 and IL-27 expression from intestinal dendritic cells which enhanced FoxP3+ T_reg_ cell number and responses [19]. Given *S. aureus*’s ability to induce IL-10 and IL-27 within the nasal tissue, this potentially implicates a role of IFN-I in *S. aureus* nasal colonization. This study, reveals that *S. aureus* can drive IFN-I production in a strain dependent manner, which ultimately induces phagocyte apoptosis as a means by which to facilitate nasal colonization.

## Results

### Gene expression analysis confirms a *S. aureus* strain dependent differential IFN-I response in bone marrow derived macrophages

**Fig. 1:**
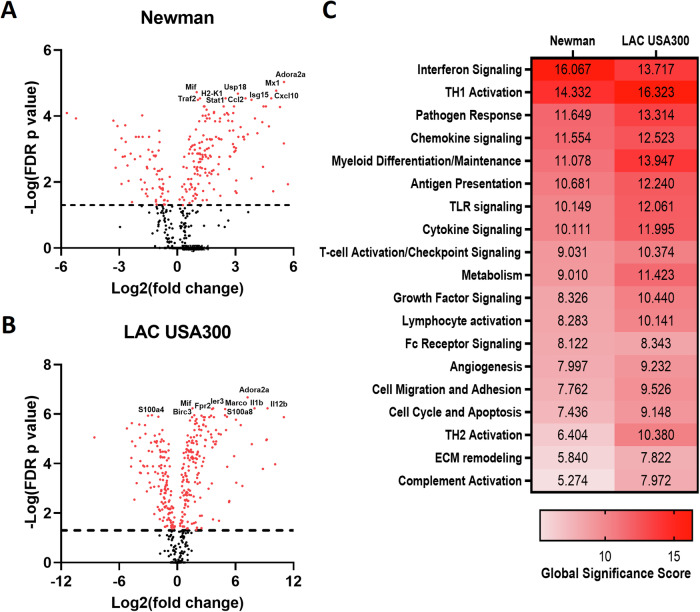
*S. aureus* elicits an IFN-I response in murine BMDMs. BMDMs were infected with *S. aureus* strains LAC USA300 or Newman at a MOI of 100 for 1 h. BMDMs were then incubated with gentamicin media for 1 h which was then replaced with antibiotic free media. At 24 h post gentamicin treatment, RNA was extracted from *S. aureus* infected BMDMs and analyzed using the myeloid panel on the Nanostring technologies nCounter system. Fold change from uninfected BMDMs was determined for Newman (**A**) and LAC USA300 (**B**) infected BMDMs using the Nanostring advanced analysis software, nSolver and graphed as volcano plots using Prism, Graphpad. Pathway analysis was also conducted for both strains (**C**) using nSolver which uses each gene’s first principal component to create a pathway score. Statistics for heatmap analysis was performed using a student’s t test comparing the means of log2 transformed normalized data. Genes with an FDR adjusted *P* value < 0.05 are highlighted red above the dotted horizontal black line.

Myeloid cells have been identified as key responders in *S. aureus* nasal colonization through the production of IL-27 and IL-10 in the NT [6]. To investigate the impact *S. aureus* has on myeloid gene expression, BMDMs were exposed to *S. aureus* strains Newman and LAC USA300 for 1 h followed by gentamicin treatment. At 24 h post gentamicin treatment, cells were lysed for RNA isolation and assessed using multiplex gene expression analysis with the Nanostring nCounter platform using the myeloid panel. Several IFN-I associated, and interferon stimulated genes (ISGs) were found among the top 10 DEGs of Newman-exposed BMDMs (*mx1*, *usp18*, *cxcl10*, *ccl2*, *isg15*, *h2-k1* and *stat1*) (Fig. 1A). Additionally, Newman drove the upregulation of the apoptosis related gene, *traf2*, and the proinflammatory mediator, *mif* [20, 21]. In contrast, LAC USA300-exposed BMDMs showed a heightened proinflammatory gene signature. The top 10 DEGs of LAC USA300-exposed BMDMs included several proinflammatory mediators (*il1b*, *il12b*, *s100a8*, *fpr2* and *mif*), the anti-apoptotic gene, *birc3*, and the phagocytic receptor, *marco* (Fig. 1B) [22–25]. Interestingly, both strains drove high expression of the adenosine receptor *adora2a*. This receptor was recently shown to enhance *S. aureus* intracellular survival within human neutrophils by suppressing the pentose phosphate pathway to limit neutrophil effector functions [26]. Overall, gene set analysis highlighted interferon signaling as a prominent feature induced by both strains, with Newman inducing the greatest number of DEGs in this pathway compared to all other gene sets (Fig. 1C).

**Fig. 2:**
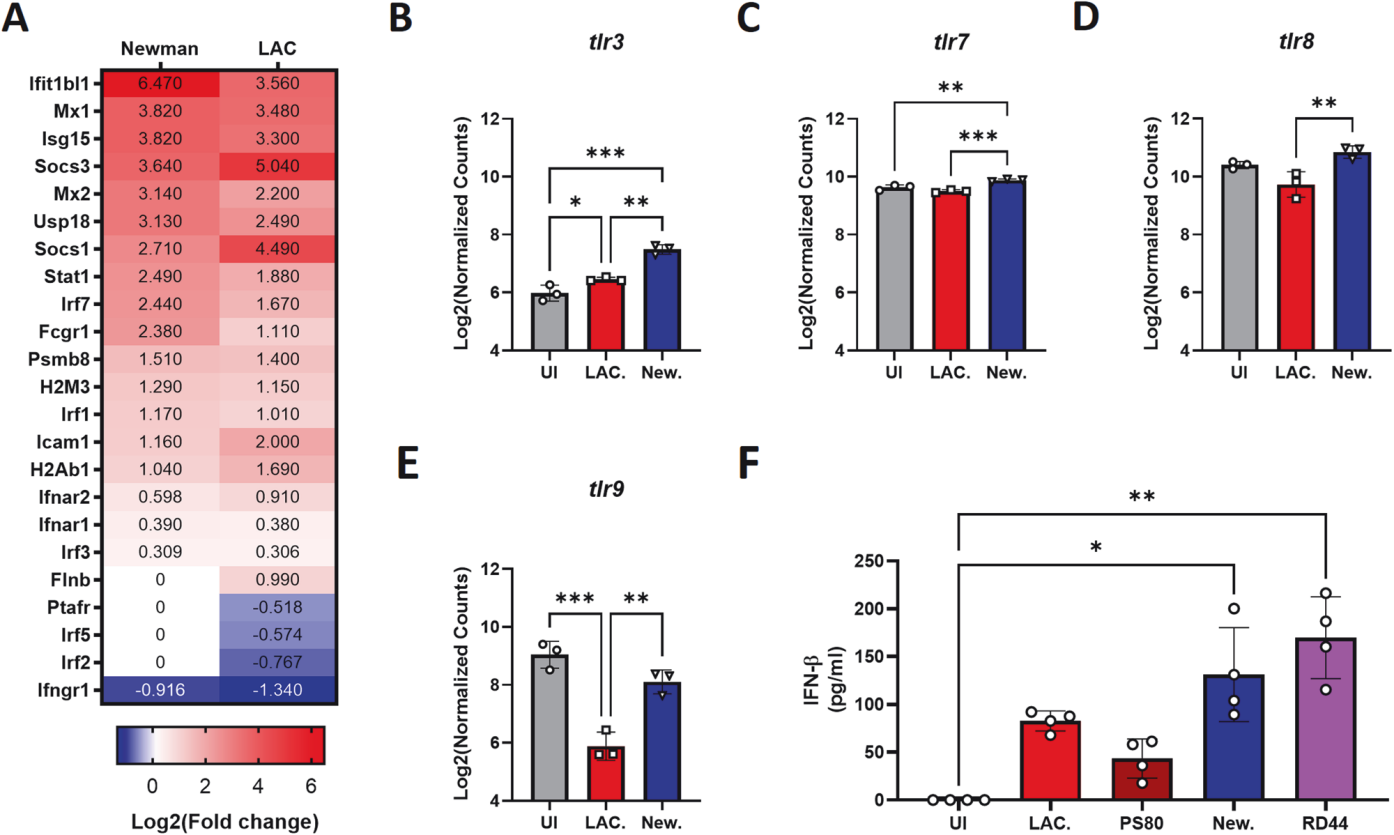
*S. aureus* strain Newman induces elevated ISGs and endosomal TLR gene expression in BMDMs compared to LAC USA300 as well as a significant increase in IFN-β secretion. Using nSolver, gene set analysis was conducted on the Nanostring gene expression analysis data from LAC USA300 or Newman infected BMDMs relative to uninfected BMDMs. Genes found in the interferon signaling gene set were then displayed using a heatmap (**A**) based on Log2(Fold change) relative to uninfected BMDMs with each gene possessing an FDR adjusted *P* value < 0.05 except for genes designated 0. TLR gene expression was assessed using Log2(normalized counts) from the Nanostring data for TLR3 (**B**), TLR7 (**C**), TLR8 (**D**) and TLR9 (**E**). BMDMs were infected with *S. aureus* strains LAC USA300, Newman, PS80 or human colonizing strain RD44 at a MOI of 100 for 1 h. BMDMs were then incubated with gentamicin media for 1 h which was then replaced with antibiotic free media. At 24 h post gentamicin treatment, cell supernatants were harvested and assessed for IFN-β secretion by ELISA (**F**). Data expressed using the mean ± SD and statistically analyzed using a one-way ANOVA with a Tukey post-test for Log2(normalized counts) or a Kruskal–Wallis test for ELISA data (*P* value * <0.05, ** <0.01, *** <0.001 and **** <0.0001).

The individual genes found within the interferon gene set were assessed to observe differences between Newman- and LAC USA300-exposed BMDMs. Newman induced an enhanced ISG signature compared to LAC USA300-exposed BMDMs (Fig. 2A). This is potentially due to the elevated expression of *socs1* and *socs3* induced by LAC USA300 which act as inhibitors of IFN-I signaling. Interestingly, Newman was also found to induce heightened gene expression of endosomal TLRs *tlr3* (Fig. 2B), *tlr7* (Fig. 2C), *tlr8* (Fig. 2D) and *tlr9* (Fig. 2E) compared to LAC USA300, which are known to drive IFN-I expression.

*S. aureus* strain Newman is commonly used in mouse models of nasal colonization due to its robust colonizing capabilities [6, 27–30]. In comparison, LAC USA300, is an invasive community acquired methicillin resistant *S. aureus* (MRSA) strain which unlike Newman, was found to be highly lethal in a rabbit sepsis model [31]. It was thus theorized that colonizing strains of *S. aureus* may induce higher IFN-I compared to more invasive strains. To test this, IFN-β was measured in LAC USA300- or Newman-exposed BMDMs and additionally to PS80, another *S. aureus* strain commonly used to model infection, or a human colonizing strain isolated from a persistent nasal carrier (RD44) [32–34]. Each strain showed some level of IFN-β secretion (Fig. 2F) possibly driven by endosomal signaling of TLR2 which has previously been shown to induce IFN-I in *S. aureus*-exposed THP-1 cells in an endocytosis dependent manner [35]. Nanostring data revealed LAC USA300 and Newman-exposed BMDMs both showed significant upregulation of TLR2 (Supplementary Fig. 1). However, Newman and RD44 showed substantially greater induction of IFN-I compared to the other strains (Fig. 2F) potentially due to enhanced expression of the other endosomal TLRs, as was observed in response to Newman (Fig. 2C–E).

To confirm IFN-I signaling in BMDMs in response to *S. aureus* strain Newman, the downstream IFN-I signaling protein STAT1 was assessed 24 h post gentamicin treatment by western blotting for both phosphorylated (pSTAT1) and total-STAT1 using WT BMDMs and BMDMs lacking the IFN-I signaling receptor (IFNAR^−/−^). Newman was found to drive total STAT1 and pSTAT1 in WT BMDMs compared to uninfected BMDMs but this was lost in IFNAR^−/−^ BMDMs (Supplementary Fig. 2), validating that Newman induced IFN-I signaling leads to STAT1 activation. In all, this data suggests that colonizing strains of *S. aureus* may be particularly adept at driving IFN-I mediated immune responses.

### Newman is less adept at intracellular survival than LAC USA300 in BMDMs but induces apoptosis in an IFN-I dependent manner

**Fig. 3:**
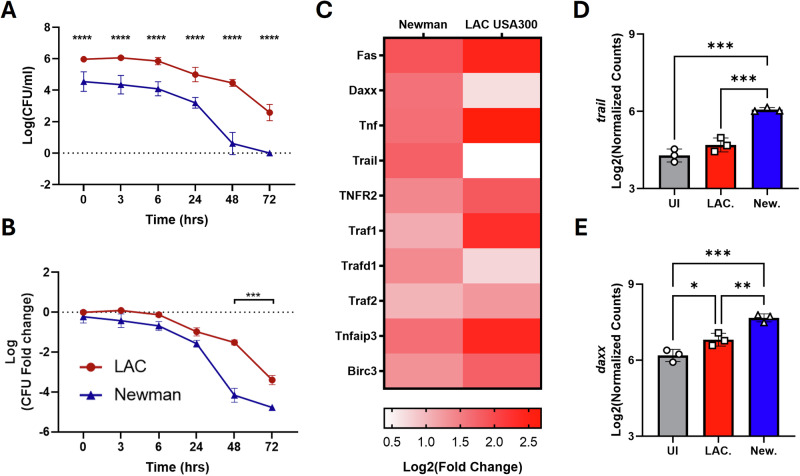
*S. aureus* strain Newman shows significantly less intracellular survival and drives the expression of the proapoptotic genes *trail* and *daxx* in BMDMs. BMDMs were infected with *S. aureus* strain LAC USA300 or Newman at a MOI of 100 for 1 h. BMDMs were then incubated with gentamicin media for 1 h which was then replaced with antibiotic free media. 24 h post gentamicin treatment, cells were then lysed for CFU enumeration and presented as either Log(CFU/ml) (**A**) or fold change of Log(CFU/ml) at T0h (**B**). Cells were lysed for RNA isolation and then assessed using the Nanostring nCounter platform with the myeloid panel. Heatmap of extrinsic apoptosis related genes contained within the Nanostring panel (**C**). Log2(Normalized counts for *trail* (**D**) and *daxx* (**E**). Results are expressed as mean ± SD with CFU data statistically analyzed using a two-way ANOVA with a Šidák multiple comparisons posttest or for the Nanostring data using a one-way ANOVA with a Tukey post-test (*P* value * <0.05, ** <0.01, *** <0.001 and **** <0.0001) for *n* = 3–6 independent experiments.

Previous studies have suggested an inverse relationship between IFN-I and *S. aureus* intracellular survival [15, 36]. As such, intracellular survival was assessed in BMDMs harboring LAC USA300 and Newman at specific time points post-gentamicin treatment. LAC USA300 displayed significantly greater levels of intracellular CFU compared to Newman at all time points (Fig. 3A). However, both strains displayed a similar trend in clearance by BMDMs and so to determine if the rate of intracellular survival differed between the strains, fold change from T0 was determined. Whilst the early infection dynamics showed a similar pattern, after 24 h post gentamicin treatment, Newman displayed significantly greater fold changes compared to LAC USA300 (Fig. 3B). This implies Newman has a reduced intracellular fitness compared to LAC USA300, which is more resistant to intracellular killing. Additionally, Newman showed a reduced rate of phagocytic uptake compared to LAC USA300 (Supplementary Fig. 3). Together, this data implicates an inverse relationship between intracellular habitation and IFN-I induction.

During listeria infection, IFN-I can drive extrinsic apoptosis in lymphocytes and macrophages to enhance persistence and bacterial proliferation [37]. Whilst *S. aureus* strain Newman was identified as a potent IFN-I inducing strain, Nanostring analysis also highlighted the upregulation of the extrinsic pro-apoptotic related gene *traf2*, potentially implicating the induction of extrinsic apoptosis in response to Newman. In contrast to this, LAC USA300 induced the anti-apoptotic *birc3* (cIAP2) (Fig. 1B) which aligns with its increased propensity to survive intracellularly and the observation that LAC USA300 can delay phagocyte apoptosis [33]. A panel of extrinsic apoptosis related genes from the Nanostring myeloid panel were therefore assessed from Newman- and LAC USA300-exposed BMDMs. LAC USA300 exposure induced enhanced upregulation of *tnf* and the proinflammatory TNF receptor, *tnfr2*, as well as the downstream signaling molecule *traf1* indicating a heightened proinflammatory TNF response rather than extrinsic apoptosis (Fig. 3C). Newman-exposed BMDMs showed a weaker TNF response but showed a significant upregulation of the proapoptotic factors *trail* and *daxx* compared to uninfected and LAC USA300-exposed BMDMs (Fig. 3D, E). BMDMs were confirmed to substantially upregulate the TRAIL receptor, TRAIL-R2, in response to *S. aureus* with Newman and the human colonizing strain RD44, showing significant upregulation compared to uninfected BMDMs (Supplementary Fig. 4).

**Fig. 4:**
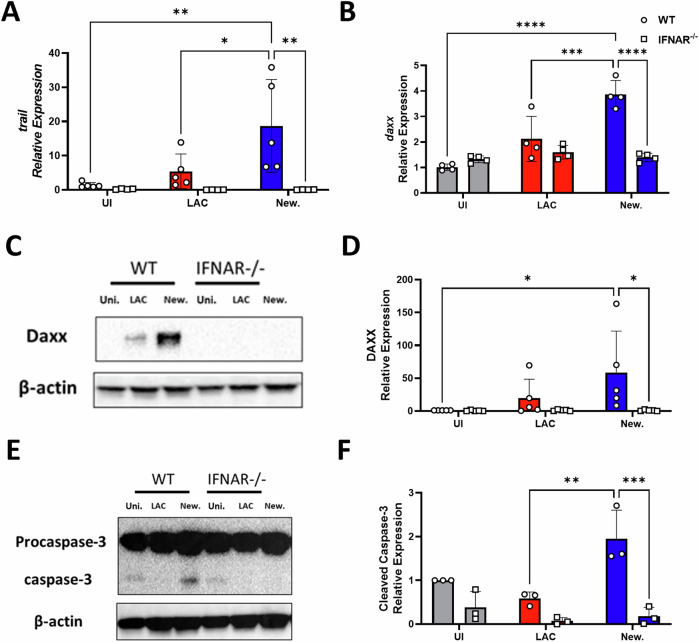
*S. aureus* strain Newman drives the expression of proapoptotic factors *trail* and *daxx* in an IFN-I dependent manner. BMDMs from WT or IFNAR^−/−^ mice were infected with *S. aureus* strains Newman or LAC USA300 at a MOI of 100 for 1 h. BMDMs were then incubated with gentamicin media for 1 h which was then replaced with antibiotic free media. 24 h post gentamicin treatment, cells were lysed for RNA extraction. Expression of the proapoptotic genes *trail* (**A**) and *daxx* (**B**) was assessed using RT-PCR in both WT and IFNAR^−/−^ mice. RT-PCR data was normalized using b2m as a housekeeping gene and analyzed using the ΔΔCT method. Protein lysates were collected 24 h post gentamicin treatment and analyzed using western blotting for Daxx (**C**) or caspase-3 (**E**) with β-actin serving as a loading control. Densitometry of Daxx (**D**) and cleaved caspase-3 (**F**) was determined using Bio-Rad’s Image Lab software, normalized to the loading control, & expressed as relative expression. Results are expressed as mean ± SD and statistically analyzed using a two-way ANOVA with a Šidák multiple comparisons posttest (*P* value * <0.05, ** <0.01, *** <0.001 and **** <0.0001) for *n* = 3–6 independent experiments.

To determine whether Newman induced *trail* and *daxx* expression was IFN-I dependent, BMDMs from IFN-receptor deficient (IFNAR^−/−^) mice, were exposed to Newman or LAC USA300, and lysed for RNA isolation. Newman induced *trail* and *daxx* was confirmed in WT BMDMs using RT-PCR and was substantially greater than uninfected or LAC USA300-exposed BMDMs (Fig. 4A, B). However, Newman failed to induce *trail* and *daxx* in IFNAR^−/−^ BMDMs indicating an IFN-I dependent response. Additionally, the IFN-I dependent induction of Daxx in response to Newman exposure was validated at the protein level by western blotting (Fig. 4C, D). Importantly, Newman was found to induce BMDM apoptosis in WT BMDMs as Newman-exposed BMDMs were found to drive significant caspase-3 cleavage compared to WT uninfected or LAC USA300-exposed BMDMs (Fig. 4E, F). However, Newman-exposed IFNAR^−/−^ BMDMs failed to induce caspase-3 cleavage confirming that Newman induced apoptosis is IFN-I dependent.

### Induction of phagocyte apoptosis in the NT facilitates *S. aureus* nasal colonization

**Fig. 5:**
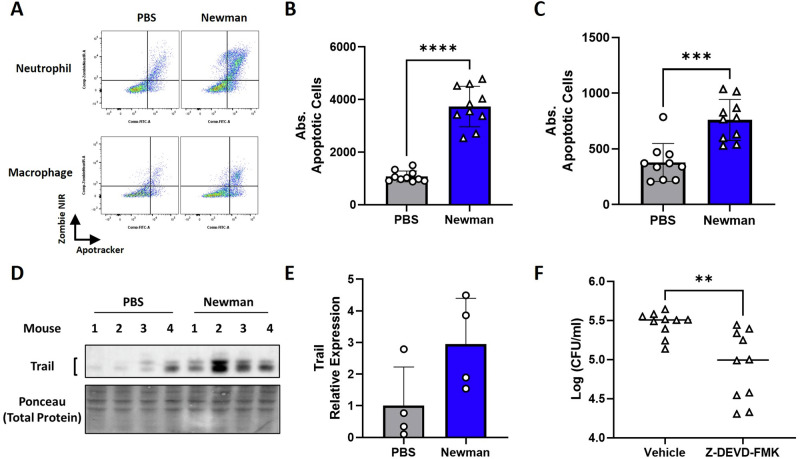
*S. aureus* induced apoptosis of neutrophils and macrophages in the nasal tissue enhances nasal colonization. Mice were colonized with streptomycin resistant Newman (2 × 10^8^ CFU/nose) and culled 24 h post-colonization to excise the NT. Cells were isolated from the nasal tissue and sequentially stained with Apotracker, Zombie NIR and extracellular markers to identify neutrophils (CD11b^+^LY6G^high^) and macrophages (CD11b^+^LY6G^low-mid^F4/80^+^) via flow cytometry. Apotracker/Zombie NIR double positive cells are deemed apoptotic cells. Results are shown as Zombie NIR vs apotracker gated on neutrophils and macrophages (**A**) with a representative plot and pooled absolute counts of apoptotic cells shown for neutrophils (**B**) and macrophages (**C**). At 24 h post-colonization, NT was excised, homogenized, and incubated with Sepharose beads to extract proteins for western blotting for Trail and total protein by Ponceau S staining (**D**). Densitometry of Trail was determined using Bio-Rad’s Image Lab software, normalized to the total protein, and expressed as relative expression (**E**). Bacterial burden within the NT was also assessed in mice that received the caspase-3 inhibitor Z-DEVD-FMK (2.5 mg/kg) compared to the vehicle control (**F**). Results are expressed as mean ± SD and statistically analyzed for animal experiments (10 mice per group) using an unpaired T test for CFU assessment and a Mann–Whitney U test for absolute counts of apoptotic cells (*P* value ** <0.01, *** <0.001 and **** <0.0001).

To determine if apoptosis of NT phagocytes played a role in nasal colonization, C57BL/6 mice were colonized with streptomycin-resistant Newman as previously described [6] and culled 24 h postnasal colonization to excise NT for flow cytometry. Neutrophils were identified by gating on CD11b^+^LY6G^hi^ cells whilst macrophages were identified by gating on CD11b^+^LY6G^low^F4/80^+^ cells (Supplementary Fig. 5a). Apoptosis was assessed in these populations using the phosphatidylserine binding dye, apotracker, and the viability dye, zombie NIR, with double positive cells representing apoptotic cells. Colonization with *S. aureus* was found to drive significant increases in the numbers of apoptotic neutrophils and macrophages within the NT compared to PBS mice (Fig. 5A–C). Percentage of apoptotic cells were also significantly enhanced by Newman nasal colonization (Supplementary Fig. 5b). Additionally, western blotting of NT homogenates revealed *S. aureus*-colonized mice expressed substantially elevated levels of Trail 24 h post colonization (Fig. 5D, E).

To ascertain how phagocytic cell apoptosis impacted *S. aureus* nasal colonization, mice were intranasally inoculated with streptomycin-resistant Newman in combination with the caspase-3 inhibitor, Z-DEVD-FMK (2.5 mg/kg), or with the DMSO vehicle control. Inhibition of caspase-3 significantly reduced bacterial burden within the NT 24 h post colonization compared to the vehicle control implicating phagocyte apoptosis leads to enhanced nasal colonization (Fig. 5F). In all, *S. aureus* was found to induce phagocyte apoptosis in the NT enhancing nasal colonization likely through the removal of these antimicrobial immune cells.

### Loss of IFN-I signaling dramatically reduces phagocytic cell apoptosis and significantly decrease nasal colonization

**Fig. 6:**
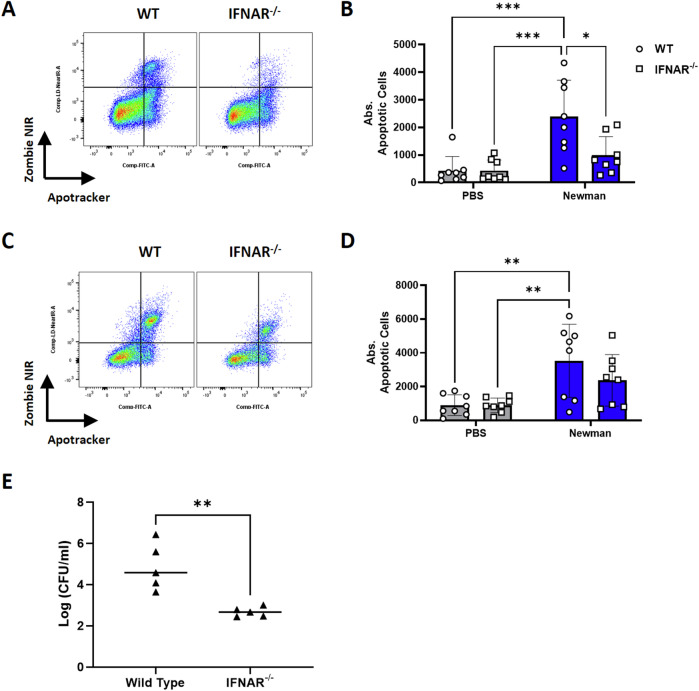
IFN-I enhances nasal colonization and drives the induction of neutrophil and macrophage apoptosis. WT and IFNAR^−/−^ mice were colonized with streptomycin resistant Newman (2 × 10^8^ CFU/nose). At 24 h post-colonization, the NT was excised and digested for flow cytometry. Cells isolated from the nasal tissue were sequentially stained with Apotracker, Zombie NIR and extracellular markers to identify neutrophils (CD11b^+^LY6G^high^) and macrophages (CD11b^+^LY6G^low-mid^F4/80^+^) via flow cytometry. Apotracker/Zombie NIR double positive cells are deemed apoptotic cells. Results are shown as Zombie NIR vs apotracker gated on neutrophils (**A** and **B**) and macrophages (**C** and **D**) with a representative plots and pooled absolute counts of apoptotic cells. On day 7, WT and IFNAR^−/−^ mice were culled to excise and homogenize the nasal tissue (**E**) for CFU enumeration. Data is expressed as mean ± SD and statistically assessed using a two-way ANOVA with a Šidák multiple comparisons post-test to compare absolute counts of late apoptotic cells or using an unpaired T test for CFU enumeration (*P* value * <0.05, ** <0.01 and *** <0.001).

In the NT, *S. aureus*-induced phagocyte apoptosis which enhanced nasal colonization. To determine if this induction of phagocytic cell apoptosis was IFN-I dependent, WT and IFNAR^−/−^ mice were intranasally colonized with streptomycin-resistant Newman. 24 h post colonization, NT was excised and enzymatically digested for flow cytometry gating on neutrophil/macrophage populations as previously described. As expected, *S. aureus*-induced apoptosis in neutrophils and macrophages in NT compared to PBS mice (Fig. 6A–D). However, *S. aureus-*induced neutrophil and macrophage apoptosis was substantially reduced in IFNAR^−/−^ mice. Importantly this reduction in phagocyte apoptosis was associated with enhanced clearance of *S. aureus* from the NT (Fig. 6E) day 7 post colonization.

## Discussion

The role of IFN-I in *S. aureus* infection is a divisive topic with both beneficial and detrimental effects reported. IFN-I contribute to protective immunity during *S. aureus* sub-cutaneous infection and post hemorrhage pneumonia with IFN-α shown to protect human lung epithelial cells from α-hemolysin-induced cell death [38–40]. Conversely, other studies have shown IFN-I contributes to *S. aureus* pathogenicity by enhancing lung damage during pneumonia and increasing mortality [15]. One possible explanation for these conflicting reports is the potential for strain dependent induction of IFN-I as well as the potential for tissue-dependent effects of IFN-I [15, 36]. For instance, the high IFN-I inducing strain 502a was responsible for exacerbating IFN-I dependent lung pathology [15]. Meanwhile, IFN-I was protective against the weaker IFN-I inducer, LAC USA300, suggesting the concentration and/or site of infection is a key determinant of the pathogenicity of IFN-I [14, 15]. However, the role of IFN-I in *S. aureus* colonization is currently unknown. This study shows that *S. aureus* indeed drives IFN-I in a strain dependent manner, with colonizing strains of *S. aureus* more potent IFN-I inducers than invasive strains. IFN-I was found to drive proapoptotic factors and apoptosis in phagocytes. It appears that during colonization, *S. aureus* drives IFN-I, leading to the induction of phagocyte apoptosis to counteract the antimicrobial effects of these cells facilitating its persistence within this niche.

Myeloid cells have previously been identified as key cells targeted by *S. aureus* to create an immunosuppressive environment within the NT [6]. Here, it is revealed that induction of IFN-I production by myeloid cells is an additional strategy employed by *S. aureus* to manipulate these cells during colonization. Gene expression analysis of BMDMs exposed to *S. aureus* strains Newman and LAC USA300 highlighted the robust induction of ISGs and interferon signaling-related genes. Interestingly, Newman showed the greatest induction of IFN-I, showing substantial upregulation of several IFN-I stimulated genes compared to LAC USA300. Newman was also shown to significantly upregulate almost all endosomal TLRs compared to LAC USA300-exposed BMDMs, indicating that the enhanced induction of IFN-I is potentially driven via increased endosomal TLR activation. This is likely due to the recent observation that Newman can drive IFN-I by releasing nucleic acid containing membrane vesicles that enter macrophages via endocytosis and leads to endosomal signaling [41]. LAC USA300 showed significantly enhanced intracellular survival and uptake compared to Newman but didn’t drive as strong an IFN-I response. This suggests that LAC USA300 may prioritize strategies to maintain an intracellular lifestyle above the induction of IFN-I as its preferred immune evasion strategy. As such, future studies should aim to address the relationship of intracellular survival and IFN-I.

Previous work has shown that *S. aureus* creates an immunosuppressive environment within the NT to facilitate persistence during colonization via IL-10/IL-27 production in tissue-resident myeloid cells [6]. LPS induction of IFN-I drives IL-27 and consequently IL-10, so it is likely that the induction of IFN-I during nasal colonization may also act to amplify the IL-10/IL-27 axis [42]. However, *S. aureus* driven IL-10 production by BMDMs is not exclusively IFN-I dependent (Supplementary Fig. 6) indicating that *S. aureus* may be exploiting IFN-I for additional means during nasal colonization. In addition to sensing/responding to *S. aureus* through the production of pro/anti-inflammatory mediators, tissue-resident and recruited phagocytes are essential for the eradication of *S. aureus* from infected tissues. Consequently, *S. aureus* exerts a significant effort to circumvent or evade the effects of these antimicrobial cells [43–45].

An important consideration in the interplay of *S. aureus* and phagocytes during infection is the induction or suppression of phagocyte apoptosis. This cellular pathway can be beneficial to the host by containing intracellular bacteria within apoptotic bodies, which are ultimately ingested by macrophages through the process of efferocytosis [46]. As such, it is unsurprising *S. aureus* has developed a complicated relationship with apoptosis where the bacterium can drive pro/antiapoptotic phenotypes in phagocytes [47]. *S. aureus* can actively inhibit neutrophil apoptosis by disrupting autophagic flux to preserve its intracellular niche whilst creating a cellular vehicle for host dissemination [33, 48]. However, many toxins produced by *S. aureus* induce apoptosis, such as alpha toxin, despite this bacterium’s adept ability to survive intracellularly [44, 47]. *S. aureus* can drive macrophage apoptosis by producing deoxyadenosine using the adenosine synthase (AdsA) virulence factor which removes macrophages in a non-inflammatory manner [49]. This was shown to be caspase-3 dependent which when deleted, protected macrophages from deoxyadenosine whilst increasing abscess infiltration for enhanced bacterial clearance. It is clear *S. aureus* can manipulate host apoptosis in either a pro or anti-apoptotic manner to suit its context dependent needs.

This study reveals that, *S. aureus* elicited a strain- and IFN-I dependent induction of phagocyte apoptosis likely through the action of Trail and Daxx. Additionally, Newman and the human colonizing strain RD44 significantly upregulated the apoptotic death receptor Trail-R2 indicating apoptosis is potentially a feature of *S. aureus* colonization that removes phagocytes to promoting persistence. Consistent with this, in a superinfection model of Influenza A virus and *Streptococcus pneumoniae*, IFN-I induced Trail led to increased bacterial outgrowth by enhancing epithelial injury [50]. Administration of anti-Trail neutralizing antibodies enhanced bacterial clearance implicating a pathogenic role of Trail during infection. The role of Daxx during bacterial infection is even less understood but is a known ISG which is upregulated during *L. monocytogenes* infection alongside Trail coinciding with general apoptosis within the spleen [51]. This suggests that bacteria can manipulate IFN-I to drive phagocyte apoptosis for their own survival which aligns with the data presented in this study.

Here, *S. aureus* was found to drive phagocyte apoptosis to increase nasal colonization which was significantly reduced when apoptosis was inhibited, indicating that phagocyte apoptosis is beneficial to the commensal lifestyle of *S. aureus*. A limitation of this study is the role that non-phagocytic cells such as epithelial cells play during colonization. Lung airway epithelial cells have been shown to respond to *S. aureus* by producing IFN-I [52, 53] and so it is likely that these cells could also contribute to the IFN-I milieu within the nasal tissue that contributes to phagocyte apoptosis. Regardless of the cellular source of IFN-I, apoptosis of the phagocytic effector cells impedes bacterial clearance and facilitates nasal colonization thus representing the key outcome of IFN-I induction.

In summary, *S. aureus* varies in its capacity to elicit an IFN-I response in macrophages, with some strong IFN-I inducing strains, leading to significant increases in the proapoptotic *trail* and *daxx* culminating in caspase 3 cleavage, the executioner of apoptosis. In vivo, Newman drives IFN-I dependent apoptosis in host neutrophils and macrophage to potentially boost its survival and persistence in the nasal cavity. In all, IFN-I has been identified as an important mediator of nasal colonization by *S. aureus*, that reshapes the host microenvironment facilitating survival and persistence by inducing phagocyte apoptosis.

## Methods & materials

### Bacterial cell culture

*S. aureus* strains used in this study were LAC USA300 [33], Newman [30] and the human colonizing strain RD44 [6] as previously described. *S. aureus* strains were streaked from frozen stocks onto TSA plates or streptomycin supplemented TSA for streptomycin resistant Newman and incubated at 37 °C overnight. Overnight stock plates were then used to prepare bacterial inocula for subsequent experiments. Bacterial colonies were scraped from stock plates and added to PBS and adjusted to an OD_600_ of 1 corresponding to 1 × 10^9^ CFU/ml. After use, bacterial inocula was then serially diluted in PBS and plated on TSA plates to confirm CFU/ml.

For phagocytic uptake assays, bacteria were incubated with CFSE (ThermoFisher, 10 µM) for 30 min under rotation immediately prior to infection.

For in vitro experiments, a single inoculate was prepared for each strain to stimulate all control and study groups at the same time and was then repeated with fresh inocula each experimental replicate as indicated in the figure legend. For in vivo experiments, one inoculum was prepared to colonize both groups of WT and IFNAR−/− mice, again in parallel. PBS was administered to control mice immediately prior to administration of *S. aureus* to the colonized groups to limit any risk of inadvertent colonization of PBS inoculated mice. In vivo experiments were repeated a second time using a fresh inoculum to simultaneously treat all groups.

### Mice

C57BL6J wild-type and IFNAR^−/−^ mice were purchased from Charles River laboratories or bred in house at the Trinity College Dublin Comparative Medicine Unit. In vivo, experiments were carried out using age matched mice (6–12 weeks) and typically carried out 1-2 times with 4-5 mice per group and there were no exclusions. In each experiment mice were allocated to their treatment group by randomization within blocks (Nuisance variables: sex, cage location). Groups were allocated by the person performing the experiment.

### Generation of murine BMDMs and Gentamicin protection assay

Murine BMDMs were generated using previously described protocols [6, 34]. On day 6, BMDMs were seeded in 12 well plates at a density of 1 × 10^6^ cells/well (for RNA and Protein analysis) in antibiotic free cDMEM and rested overnight. BMDMs were then infected at a multiplicity of infection (MOI) of 100 for 1 h before gentamicin treatment for a further 1 h (200 µg/ml). Gentamicin containing media was removed and replaced with antibiotic free cDMEM. At indicated time points, cell supernatants were removed for ELISA and cells lysed in Qiazol for RNA isolation. Alternatively, Cells were lysed for CFU enumeration using triton-X-100 (0.1%) for 10 min followed by plating on TSA agar plates and overnight incubation at 37 °C or for western blotting by lysing on ice using 1x Sample buffer with β-mercaptoethanol.

### RNA isolation, cDNA synthesis and RT-PCR

RNA was isolated at indicated timepoints using a Qiagen (Germantown, MD, USA) RNeasy mini plus kit as per manufacturer’s instruction and then assessed for RNA concentration and quality using an LVis plate and a SpectrostarNano microplate reader. cDNA synthesis was achieved using the High-capacity cDNA reverse transcription kit (Applied Biosystems, Waltham, Massachusetts, USA) as per protocol and then stored at −20 °C.

cDNA was assessed by RT-PCR with a CFX96 touch RT-PCR system (Bio-Rad) using reverse and forward primers for *trail* (Primer sequence:) and *daxx* (Primer sequence:) alongside iTaq™ Sybr Green. Results were then exported and analyzed using Bio-Rad CFX Manager Software. Ct values were exported to excel and analyzed using the ∆∆ct method.

### Nanostring nCounter platform

For Nanostring analysis, RNA was isolated at 24 h post gentamicin treatment and assessed using a qubit fluorometer and RNA HS assay kit to validate both RNA quality and concentration. RNA (100 ng) was then assessed using the myeloid panel and the Nanostring nCounter Flex system (Seattle, Washington, USA) according to manufacturer’s protocol. Data was then exported and analyzed using nSolver version 4 with the advanced analysis software plugin. 754 myeloid associated genes were assessed per strain and expressed relative to the uninfected control. Statistically, the Log2(normalized counts) per gene from *S. aureus* exposed BMDMs was compared to uninfected control BMDMs using a Student’s *t* test. *P* values were then adjusted to account for false discoveries using a Benjamini–Yekutieli post-test and represented on a volcano plot with the top 10 most significantly differentially expressed genes (DEGs) highlighted for each strain.

### ELISA

At indicated timepoints, IL-10 and IFN-β (DuoSet ELISA, R&D Systems, Minneapolis, MN, USA) were measured in cell supernatants or nasal tissue homogenates by ELISA according to manufacturer’s guidelines.

### Western blotting

Samples were separated using 4–20% precast TGX polyacrylamide gels (BioRad) and transferred onto polyvinylidene difluoride membranes. Membranes were blocked in Advanblock (Advansta Inc, San Jose, CA) for 1 h at RT under constant agitation followed by 3 × 5 min washes in PBST. Membranes were then probed overnight with the following antibodies as indicated at 4 °C under constant agitation: rabbit anti-mouse DAXX (Cell Signaling Technology, Danvers, Massachusetts, USA), rabbit anti-mouse Caspase-3 (Cell Signaling Technology, Danvers, Massachusetts, USA), rabbit anti-mouse β-actin (Cell Signaling Technology, Danvers, Massachusetts, USA), rat anti-mouse Trail (R&D biosystems, Minneapolis, MN, USA), pSTAT1 and total STAT1 (Cell Signaling Technology, Danvers, Massachusetts, USA). Membranes were washed 3x for 5 min in PBST followed by incubation for 1 h with anti-rabbit or anti-rat secondary HRP-conjugated antibody at RT under constant agitation. Membranes were washed as before and then developed using ECL and a CCD camera (Bio-Rad Gel Dock). Data was normalized using β-actin. For in vivo samples, total protein normalization was used by ponceau S staining blots for 5 min, washing in distilled water and imaging for total protein. Uncropped blots are available in the supplemental information files.

### Murine nasal colonization model

WT or IFNAR^−/−^ mice were given sterile water containing streptomycin (0.5 mg/ml) 72 h prior to nasal colonization with continued streptomycin treatment given throughout the duration of the experiment. After streptomycin treatment, mice were intranasally administered streptomycin resistant *S. aureus* strain Newman (2 × 10^8^ CFU/nose) under isoflurane treatment. For the inhibition of caspase-3, bacterial inocula was co-administered with the caspase-3 inhibitor, Z-DEVD-FMK (2.5 mg/kg). At specific timepoints, mice were euthanized using CO_2_ exposure and the nose and NT excised as previously described [6]. The nose and NT were homogenized with a Polytron PT 2500E (Kinematica, Malters, Switzerland), serially diluted, and plated onto TSA plates containing streptomycin followed by overnight incubation at 37 °C. For western blotting, Sepharose beads (Agilent) were added to nasal tissue homogenates, briefly vortexed and incubated for 5 min on a roller. Homogenates were then centrifuged at 300 g for 5 min, supernatants discarded, beads resuspended in sample buffer and boiled for 5 min at 95 °C. Tissue lysates were then assessed for Trail using SDS-PAGE and western blotting as above.

### Cell dissociation of NT and flow cytometry

To obtain a single cell suspension, the NT was enzymatically digested using cRPMI containing Collagenase D (1 mg/ml) and DNase (0.1 mg/ml) for 30 min at 37 °C in a shaking incubator at 250 RPM. The resulting single cell suspension was then passed through a 40 μM nylon Falcon cell strainer and then centrifuged for 5 min at 300 g followed by red blood cell lysis using ACK lysis buffer. Cells were washed and centrifuged for 5 min at 300 g, resuspended and used immediately for flow cytometry staining.

Cells were washed with PBS and incubated for 15 min with apotracker (200 nM), a cell impermeable phosphatidyl serine binding dye. After this, cells were washed and incubated with Zombie Near IR fixable viability dye for 15 min followed by 15 min incubation with mouse Fc Receptor Binding Inhibitor Polyclonal Antibody. Cells were washed in PBS containing 1% bovine serum albumin. Cells were extracellularly stained for 15 min and then fixed with FIX & PERM™ fix A (Thermofisher, Waltham, MA, USA) for 15 min. Samples were assessed using a BD FACSCanto II (BD biosciences, San Jose, CA, USA) cytometer. Apoptosis was assessed by first gating of the population of interest and then plotting Zombie Near IR against apotracker with double positive cells defined as late apoptotic with single positive cells for Zombie Near IR or apotracker defined as necrotic and early apoptotic respectively.

### Statistical analysis

Prism GraphPad was used to perform statistical analysis of all the data generated except for the Nanostring data which was as previously described. A one-way ANOVA with a Tukey’s post-test comparison was used for multiple groups compared at one time point. In animal experiments, an ordinary Student’s *t* test was employed for log transformed CFU data or a Mann–Whitney U test for comparison of absolute cell numbers. Groups compared across several variables were assessed using a two-way ANOVA with a Šídák’s correction posttest. *P* values less than 0.05 were considered statistically significant. Power calculations were generated using G-power software. Calculations based on previously published studies using models of *S. aureus* colonization [6, 30].

## Supplementary information

Supplemental material

## Data Availability

All data generated or analyzed during this study are included in this published article and its supplementary information.

## References

[1] Kluytmans J, Van Belkum A, Verbrugh H. Nasal carriage of Staphylococcus aureus: Epidemiology, underlying mechanisms, and associated risks. Clin Microbiol Rev. 1997;10:505–20. 9227864 10.1128/CMR.10.3.505 PMC172932

[2] Sakr A, Brégeon F, Mège JL, Rolain JM, Blin O. Staphylococcus aureus nasal colonization: An update on mechanisms, epidemiology, risk factors, and subsequent infections. Front Microbiol. 2018;9:2419. 30349525 10.3389/fmicb.2018.02419 PMC6186810

[3] Garzoni C, Kelley WL. Return of the Trojan horse: Intracellular phenotype switching and immune evasion by Staphylococcus aureus. EMBO Mol Med. 2011;3:115–7. https://pubmed.ncbi.nlm.nih.gov/21365763/. 21365763 10.1002/emmm.201100123 PMC3395108

[4] Tuchscherr L, Medina E, Hussain M, Völker W, Heitmann V, Niemann S, et al. Staphylococcus aureus phenotype switching: An effective bacterial strategy to escape host immune response and establish a chronic infection. EMBO Mol Med. 2011;3:129–41. https://onlinelibrary.wiley.com/doi/abs/10.1002/emmm.201000115. 21268281 10.1002/emmm.201000115 PMC3395110

[5] Leech JM, Lacey KA, Mulcahy ME, Medina E, McLoughlin RM. IL-10 Plays Opposing Roles during Staphylococcus aureus Systemic and Localized Infections. J Immunol. 2017;198:2352–65. 28167629 10.4049/jimmunol.1601018 PMC5337812

[6] Kelly AM, Leech JM, Doyle SL, McLoughlin RM. Staphylococcus aureus-induced immunosuppression mediated by IL-10 and IL-27 facilitates nasal colonisation. PLoS Pathog. 2022;18:e1010647. 35776778 10.1371/journal.ppat.1010647 PMC9282462

[7] Mulcahy ME, Leech JM, Renauld JC, Mills KHG, McLoughlin RM. Interleukin-22 regulates antimicrobial peptide expression and keratinocyte differentiation to control Staphylococcus aureus colonization of the nasal mucosa. Mucosal Immunol. 2016;9:1429–41. http://www.mucosalimmunology.org/article/S1933021922007863/fulltext. 27007677 10.1038/mi.2016.24

[8] Archer NK, Adappa ND, Palmer JN, Cohen NA, Harro JM, Lee SK, et al. Interleukin-17A (IL-17A) and IL-17F Are Critical for Antimicrobial Peptide Production and Clearance of Staphylococcus aureus Nasal Colonization. Infect Immun. 2016;84:3575. 27736775 10.1128/IAI.00596-16 PMC5116734

[9] Archer NK, Harro JM, Shirtliff ME. Clearance of Staphylococcus aureus nasal carriage is T cell dependent and mediated through interleukin-17A expression and neutrophil influx. Infect Immun. 2013;81:2070–5. https://pubmed.ncbi.nlm.nih.gov/23529621/. 23529621 10.1128/IAI.00084-13 PMC3676016

[10] Schulz A, Jiang L, De Vor L, Ehrströ M, Wermeling F, Eidsmo L, et al. Neutrophil Recruitment to Noninvasive MRSA at the Stratum Corneum of Human Skin Mediates Transient Colonization. CellReports. 2019;29:1074–81. 10.1016/j.celrep.2019.09.055. 10.1016/j.celrep.2019.09.055 31665625

[11] McNab F, Mayer-Barber K, Sher A, Wack A, O’Garra A. Type I interferons in infectious disease. Nat Rev Immunol. 2015;15:87–103. https://www.nature.com/articles/nri3787. 25614319 10.1038/nri3787 PMC7162685

[12] Carrero JA, Calderon B, Unanue ER. Type I interferon sensitizes lymphocytes to apoptosis and reduces resistance to Listeria infection. J Exp Med. 2004;200:535–40. 15302900 10.1084/jem.20040769 PMC2211931

[13] Costa Franco MM, Marim F, Guimarães ES, Assis NRG, Cerqueira DM, Alves-Silva J. et al. Brucella abortus Triggers a cGAS-Independent STING Pathway To Induce Host Protection That Involves Guanylate-Binding Proteins and Inflammasome Activation. J Immunol. 2018;200:607–22. 10.4049/jimmunol.1700725. 29203515 10.4049/jimmunol.1700725 PMC5760291

[14] Klopfenstein N, Brandt SL, Castellanos S, Gunzer M, Blackman A, Serezani CH. SOCS-1 inhibition of type I interferon restrains Staphylococcus aureus skin host defense. PLoS Pathog. 2021;17:e1009387. 33690673 10.1371/journal.ppat.1009387 PMC7984627

[15] Parker D, Planet PJ, Soong G, Narechania A, Prince A. Induction of Type I Interferon Signaling Determines the Relative Pathogenicity of Staphylococcus aureus Strains. Kielian T, editor. PLoS Pathog. 2014;10:e1003951. 10.1371/journal.ppat.1003951. 24586160 10.1371/journal.ppat.1003951 PMC3930619

[16] Spolski R, West EE, Li P, Veenbergen S, Yung S, Kazemian M, et al. IL-21/type I interferon interplay regulates neutrophil-dependent innate immune responses to Staphylococcus aureus. Elife. 2019;8:45501. https://elifesciences.org/articles/45501. 10.7554/eLife.45501 PMC6504231 30969166

[17] Sirobhushanam S, Parsa N, Reed TJ, Berthier CC, Sarkar MK, Hile GA, et al. Staphylococcus aureus Colonization Is Increased on Lupus Skin Lesions and Is Promoted by IFN-Mediated Barrier Disruption. J Investigative Dermatol. 2020;140:1066–74. e4 10.1016/j.jid.2019.11.016 PMC7183889 31877319

[18] Postal M, Vivaldo JF, Fernandez-Ruiz R, Paredes JL, Appenzeller S, Niewold TB. Type I interferon in the pathogenesis of systemic lupus erythematosus. Curr Opin Immunol. 2020;67:87. 33246136 10.1016/j.coi.2020.10.014 PMC8054829

[19] Vasquez Ayala A, Hsu CY, Oles RE, Matsuo K, Loomis LR, Buzun E, et al. Commensal bacteria promote type I interferon signaling to maintain immune tolerance in mice. J Exp Med. 2024;221:e20230063. 38085267 10.1084/jem.20230063 PMC10716256

[20] Siegmund D, Wagner J, Wajant H. TNF Receptor Associated Factor 2 (TRAF2) Signaling in Cancer. Cancers. 2022;14:4055. https://www.mdpi.com/2072-6694/14/16/4055/htm. 36011046 10.3390/cancers14164055 PMC9406534

[21] Sumaiya K, Langford D, Natarajaseenivasan K, Shanmughapriya S. Macrophage migration inhibitory factor (MIF): A multifaceted cytokine regulated by genetic and physiological strategies. Pharm Ther. 2022;233:108024. 10.1016/j.pharmthera.2021.108024 34673115

[22] Orecchioni M, Ghosheh Y, Pramod AB, Ley K. Macrophage polarization: Different gene signatures in M1(Lps+) vs. Classically and M2(LPS-) vs. Alternatively activated macrophages. Front Immunol. 2019;10:451543. 10.3389/fimmu.2019.01084 PMC6543837 31178859

[23] Kissick HT, Dunn LK, Ghosh S, Nechama M, Kobzik L, Arredouani MS. The Scavenger Receptor MARCO Modulates TLR-Induced Responses in Dendritic Cells. Allen IC, editor. PLoS One. 2014;9:e104148. 10.1371/journal.pone.0104148. 25089703 10.1371/journal.pone.0104148 PMC4121322

[24] Wang S, Song R, Wang Z, Jing Z, Wang S, Ma J. S100A8/A9 in inflammation. Front Immunol. 2018;9:1298. 10.3389/fimmu.2018.01298 PMC6004386 29942307

[25] Park JM, Greten FR, Wong A, Westrick RJ, Arthur JSC, Otsu K, et al. Signaling pathways and genes that inhibit pathogen-induced macrophage apoptosis - CREB and NF-κB as key regulators. Immunity. 2005;23:319–29. 16169504 10.1016/j.immuni.2005.08.010

[26] Vozza EG, Daly CM, O’Rourke SA, Fitzgerald HK, Dunne A, McLoughlin RM. Staphylococcus aureus suppresses the pentose phosphate pathway in human neutrophils via the adenosine receptor A2aR to enhance intracellular survival. mBio. 2023; Available from: https://journals.asm.org/journal/mbio 10.1128/mbio.02571-23 PMC10790693 38108639

[27] Schaffer AC, Solinga RM, Cocchiaro J, Portoles M, Kiser KB, Risley A, et al. Immunization with Staphylococcus aureus clumping factor B, a major determinant in nasal carriage, reduces nasal colonization in a murine model. Infect Immun. 2006;74:2145–53. 10.1128/iai.74.4.2145-2153.2006. 16552044 10.1128/iai.74.4.2145-2153.2006 PMC1418917

[28] Holtfreter S, Radcliff FJ, Grumann D, Read H, Johnson S, Monecke S, et al. Characterization of a Mouse-Adapted Staphylococcus aureus Strain. PLoS One. 2013;8:e71142. https://journals.plos.org/plosone/article?id=10.1371/journal.pone.0071142. 24023720 10.1371/journal.pone.0071142 PMC3759423

[29] Misawa Y, Kelley KA, Wang X, Wang L, Park WB, Birtel J, et al. Staphylococcus aureus Colonization of the Mouse Gastrointestinal Tract Is Modulated by Wall Teichoic Acid, Capsule, and Surface Proteins. PLoS Pathog. 2015;11:e1005061. https://journals.plos.org/plospathogens/article?id=10.1371/journal.ppat.1005061. 26201029 10.1371/journal.ppat.1005061 PMC4511793

[30] Mulcahy ME, Geoghegan JA, Monk IR, O’Keeffe KM, Walsh EJ, Foster TJ, et al. Nasal Colonisation by Staphylococcus aureus Depends upon Clumping Factor B Binding to the Squamous Epithelial Cell Envelope Protein Loricrin. PLoS Pathog. 2012;8:e1003092. https://journals.plos.org/plospathogens/article?id=10.1371/journal.ppat.1003092. 23300445 10.1371/journal.ppat.1003092 PMC3531522

[31] Spaulding AR, Satterwhite EA, Lin YC, Chuang-Smith ON, Frank KL, Merriman JA, et al. Comparison of Staphylococcus aureus strains for ability to cause infective endocarditis and lethal sepsis in rabbits. Front Cell Infect Microbiol. 2012;2:18. 22919610 10.3389/fcimb.2012.00018 PMC3417574

[32] Verkaik NJ, De Vogel CP, Boelens HA, Grumann D, Hoogenboezem T, Vink C, et al. Anti-staphylococcal humoral immune response in persistent nasal carriers and noncarriers of Staphylococcus aureus. J Infect Dis. 2009;199:625–32. https://pubmed.ncbi.nlm.nih.gov/19199541/. 19199541 10.1086/596743

[33] Mulcahy ME, O’Brien EC, O’Keeffe KM, Vozza EG, Leddy N, McLoughlin RM. Manipulation of Autophagy and Apoptosis Facilitates Intracellular Survival of Staphylococcus aureus in Human Neutrophils. Front Immunol. 2020;11:2906. https://www.frontiersin.org/articles/10.3389/fimmu.2020.565545/full. 10.3389/fimmu.2020.565545 PMC7686353 33262756

[34] O’Keeffe KM, Wilk MM, Leech JM, Murphy AG, Laabei M, Monk IR, et al. Manipulation of Autophagy in Phagocytes Facilitates Staphylococcus aureus Bloodstream Infection. Infect Immun. 2015;83:3445–57. http://www.ncbi.nlm.nih.gov/pubmed/26099586. 26099586 10.1128/IAI.00358-15 PMC4534639

[35] Musilova J, Mulcahy ME, Kuijk MM, McLoughlin RM, Bowie AG. Toll-like receptor 2–dependent endosomal signaling by Staphylococcus aureus in monocytes induces type I interferon and promotes intracellular survival. J Biol Chem. 2019;294:17031. 31558608 10.1074/jbc.RA119.009302 PMC6851302

[36] Peignier A, Planet PJ, Parker D. Differential Induction of Type I and III Interferons by Staphylococcus aureus. Infect Immun. 2020;88:e00352–20. 32690637 10.1128/IAI.00352-20 PMC7504949

[37] Boxx GM, Cheng G. The Roles of Type i Interferon in Bacterial Infection. Cell Host and Microbe. 2016;19:760–9. 10.1016/j.chom.2016.05.016 PMC5847370 27281568

[38] Kaplan A, Ma J, Kyme P, Wolf AJ, Becker CA, Tseng CW, et al. Failure To Induce IFN-β Production during Staphylococcus aureus Infection Contributes to Pathogenicity. J Immunol. 2012;189:4537–45. 23008447 10.4049/jimmunol.1201111 PMC3478442

[39] Lizak M, Yarovinsky TO. Phospholipid scramblase 1 mediates type i interferon-induced protection against staphylococcal α-toxin. Cell Host Microbe. 2012;11:70–80. 22264514 10.1016/j.chom.2011.12.004 PMC3266557

[40] Roquilly A, Gautreau L, Segain JP, de Coppet P, Sebille V, Jacqueline C, et al. CpG-ODN and MPLA Prevent Mortality in a Murine Model of Post-Hemorrhage-Staphyloccocus aureus Pneumonia. Neyrolles O, editor. PLoS One. 2010;5:e13228. 10.1371/journal.pone.0013228. 20949109 10.1371/journal.pone.0013228 PMC2951351

[41] Rodriguez BV, Kuehn MJ. Staphylococcus aureus secretes immunomodulatory RNA and DNA via membrane vesicles. Sci Rep. 2020;10:1–22. https://www.nature.com/articles/s41598-020-75108-3. 33106559 10.1038/s41598-020-75108-3 PMC7589478

[42] Iyer SS, Ghaffari AA, Cheng G. Lipopolysaccharide-Mediated IL-10 Transcriptional Regulation Requires Sequential Induction of Type I IFNs and IL-27 in Macrophages. J Immunol. 2010;185:6599–607. 10.4049/jimmunol.1002041. 21041726 10.4049/jimmunol.1002041 PMC4103176

[43] Fraunholz M, Sinha B. Intracellular Staphylococcus aureus: live-in and let die. Front Cell Infect Microbiol. 2012;2:43. 22919634 10.3389/fcimb.2012.00043 PMC3417557

[44] Missiakas D, Winstel V. Selective Host Cell Death by Staphylococcus aureus: A Strategy for Bacterial Persistence. Front Immunol. 2021;11:3518. 10.3389/fimmu.2020.621733 PMC7859115 33552085

[45] Howden BP, Giulieri SG, Wong Fok Lung T, Baines SL, Sharkey LK, Lee JYH, et al. Staphylococcus aureus host interactions and adaptation. Nat Rev Microbiol. 2023;21:380–95. https://www.nature.com/articles/s41579-023-00852-y. 36707725 10.1038/s41579-023-00852-y PMC9882747

[46] Martin CJ, Peters KN, Behar SM. Macrophages clean up: Efferocytosis and microbial control. Curr Opin Microbiol. 2014;17:17–23. 10.1016/j.mib.2013.10.007 PMC3942671 24581688

[47] Soe YM, Bedoui S, Stinear TP, Hachani A. Intracellular Staphylococcus aureus and host cell death pathways. Cell Microbiol. 2021;23:e13317. https://onlinelibrary.wiley.com/doi/full/10.1111/cmi.13317. 33550697 10.1111/cmi.13317

[48] Vozza EG, Mulcahy ME, McLoughlin RM. Making the Most of the Host; Targeting the Autophagy Pathway Facilitates Staphylococcus aureus Intracellular Survival in Neutrophils. Front Immunol. 2021;12:2304. 10.3389/fimmu.2021.667387 PMC8242348 34220813

[49] Winstel V, Schneewind O, Missiakas D. Staphylococcus aureus exploits the host apoptotic pathway to persist during infection. mBio. 2019;10. Available from: 10.1128/mBio.02270-19 10.1128/mBio.02270-19 PMC6851280 31719177

[50] Ellis GT, Davidson S, Crotta S, Branzk N, Papayannopoulos V, Wack A. TRAIL+ monocytes and monocyte-related cells cause lung damage and thereby increase susceptibility to influenza–Streptococcus pneumoniae coinfection. EMBO Rep. 2015;16:1203. 26265006 10.15252/embr.201540473 PMC4576987

[51] O’Connell RM, Saha SK, Vaidya SA, Bruhn KW, Miranda GA, Zarnegar B, et al. Type I interferon production enhances susceptibility to Listeria monocytogenes infection. J Exp Med. 2004;200:437–45. 15302901 10.1084/jem.20040712 PMC2211937

[52] Parker D, Prince A. The type I IFN response to extracellular bacteria in the airway epithelium. Trends Immunol. 2011;32:582. 21996313 10.1016/j.it.2011.09.003 PMC3221817

[53] Martin FJ, Gomez MI, Wetzel DM, Memmi G, O’Seaghdha M, Soong G, et al. Staphylococcus aureus activates type I IFN signaling in mice and humans through the Xr repeated sequences of protein A. J Clin Investig. 2009;119:1931–9. 19603548 10.1172/JCI35879 PMC2701857

