# Supplementary material for: Type 1 interferons promote Staphylococcus aureus nasal colonization by inducing phagocyte apoptosis
Source: Cell Death Discov. 2024 Sep 13;10:403. doi: 10.1038/s41420-024-02173-2 (PMC11399434; doi:10.1038/s41420-024-02173-2)
Supplement: Supplementary file 1 — Supplemental material [file 41420_2024_2173_MOESM1_ESM.pdf]

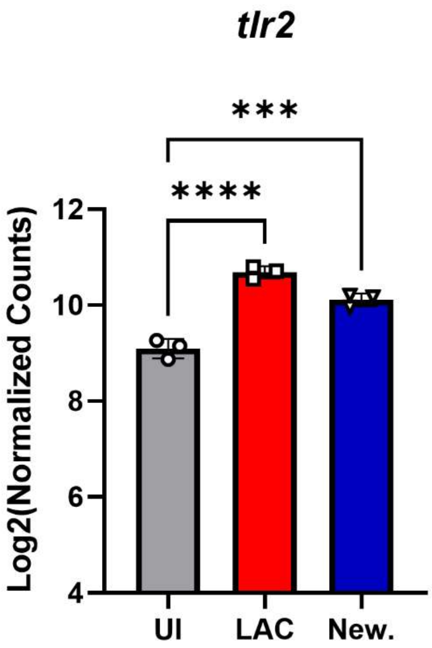

Supplemental Figure 1: *tlr2* gene expression in BMDMs infected with *S. aureus* strain LAC USA300 or Newman.

TLR gene expression was assessed using Log2(normalised counts) from the Nanostring data for TLR2. Data expressed using the mean ± SD and statistically analysed using a one-way ANOVA with a Tukey post-test for Log2(normalised counts) (*P* value *** < 0.001 and **** < 0.0001).

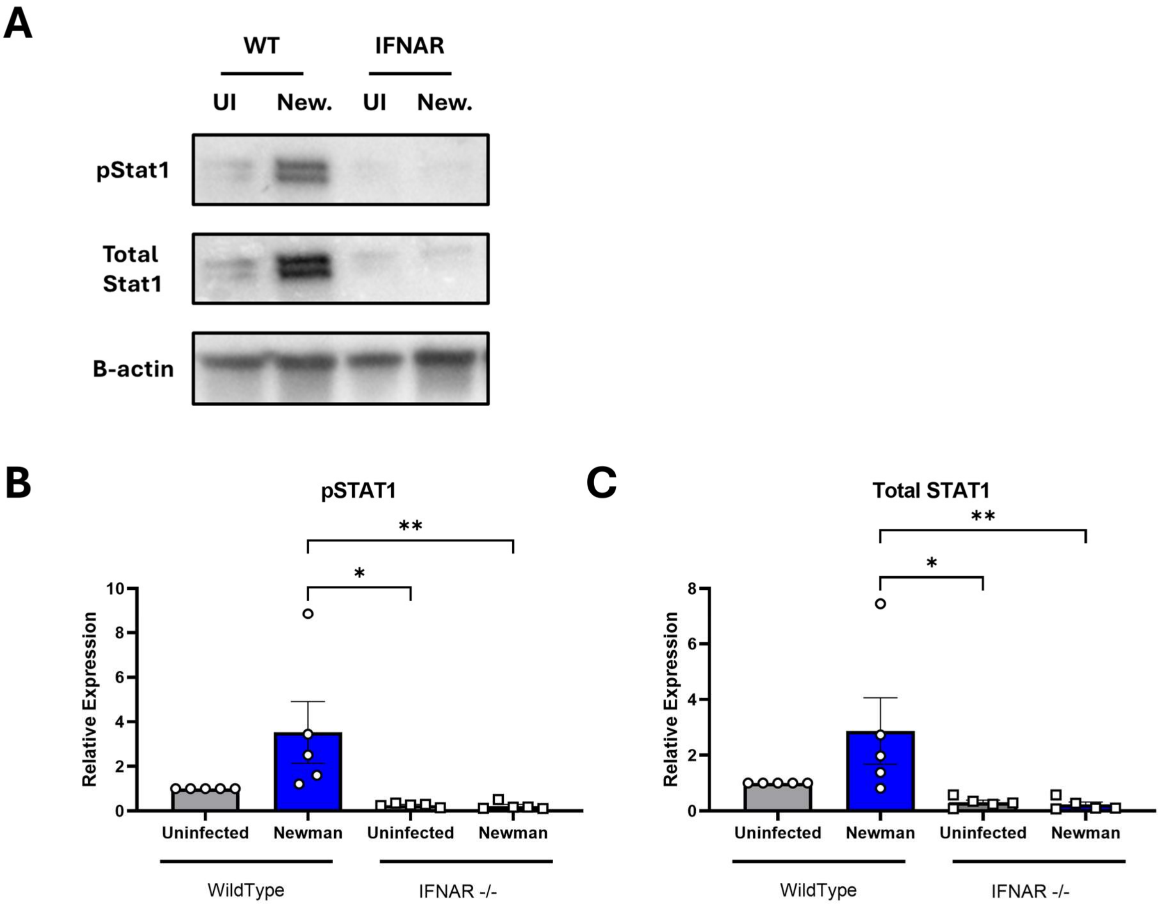

Supplemental Figure 2: Stat1 activation in WT and IFNAR$^{-/-}$ BMDMs infected with *S. aureus* strain Newman.

BMDMs were infected with *S. aureus* strain Newman at a MOI of 100 for 1 h. BMDMs were then incubated with gentamicin media for 1 h which was then replaced with antibiotic free media. Protein lysates were collected 24 h post gentamicin treatment and analysed using western blotting for phosphorylated Stat1 or total Stat1 (A) with β-actin serving as a loading control. Densitometry of phosphorylated Stat1 (B) and total Stat1 (C) was determined using Bio-Rad's Image Lab software, normalized to the loading control, & expressed as relative expression. Densitometry was statistically analysed using a Kruskal-Wallis test (*P* value * < 0.05 and ** < 0.01).

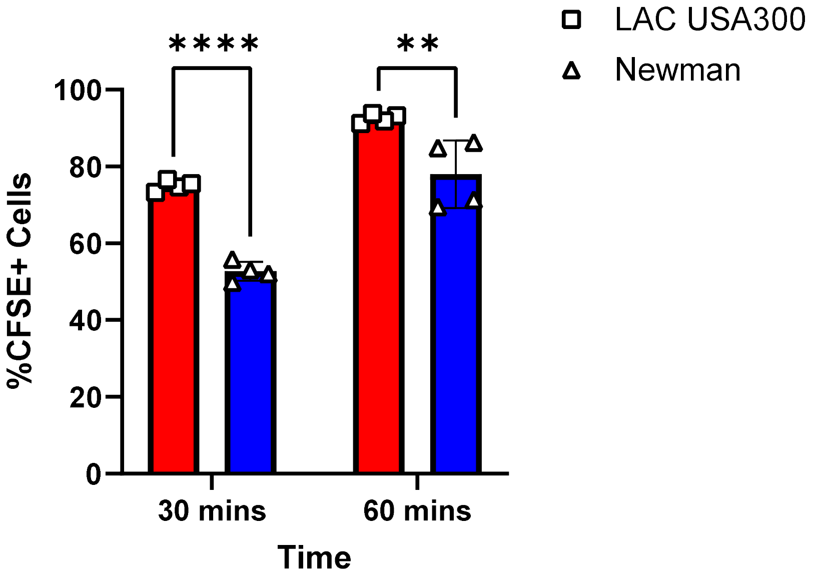

Supplemental Figure 3: Phagocytic uptake of LAC USA300 and Newman within BMDMs.

S. aureus strain LAC USA300 and Newman were incubated with CFSE (10 μM) for 30 mins under rotation to fluorescently label each strain. BMDMs were then exposed to CFSE-labelled LAC USA300 or Newman at a MOI of 100 for 30 & 60 mins. Cells were then lifted with cold PBS and vigorous pipetting followed by flow cytometric analysis. Results are display as the mean %CFSE positive cells (±SD) and statistically assessed using a two-way ANOVA with a Šidák multiple comparisons posttest (P value ** < 0.01, and **** < 0.0001) for n = 4 independent experiments.

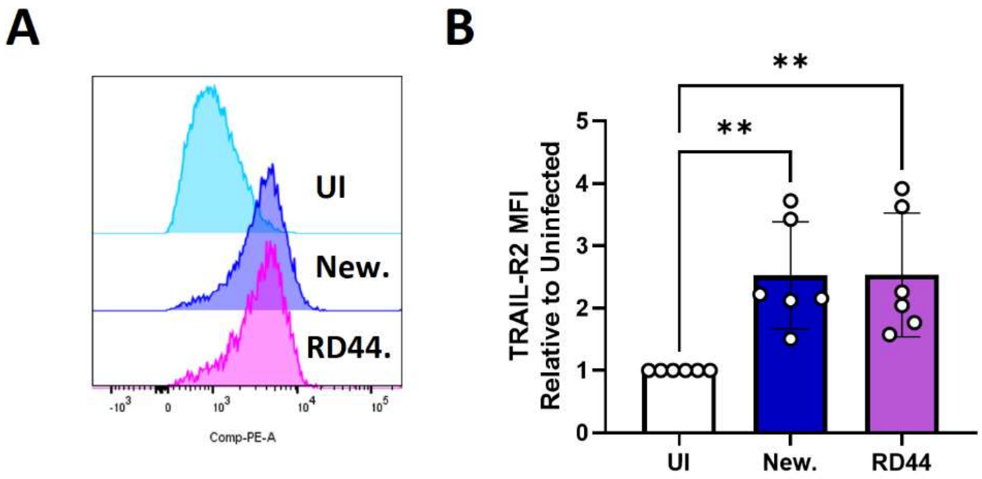

Supplemental Figure 4: TRAIL-R2 expression in BMDMs infected with *S. aureus* strain Newman or the human colonizing strain, RD44.

BMDMs were infected with *S. aureus* strain LAC USA300, Newman or RD44 at a MOI of 100 for 1 h. BMDMs were then incubated with gentamicin media for 1 h which was then replaced with antibiotic free media. 24 h post gentamicin treatment, BMDM surface expression of TRAIL-R2 was assessed by flow cytometry and displayed as a representative histogram (D) with pooled MFI data (E). Flow cytometric data was statistically analysed using a Kruskal-Wallis test (*P* value * < 0.05, ** < 0.01 and *** < 0.001)

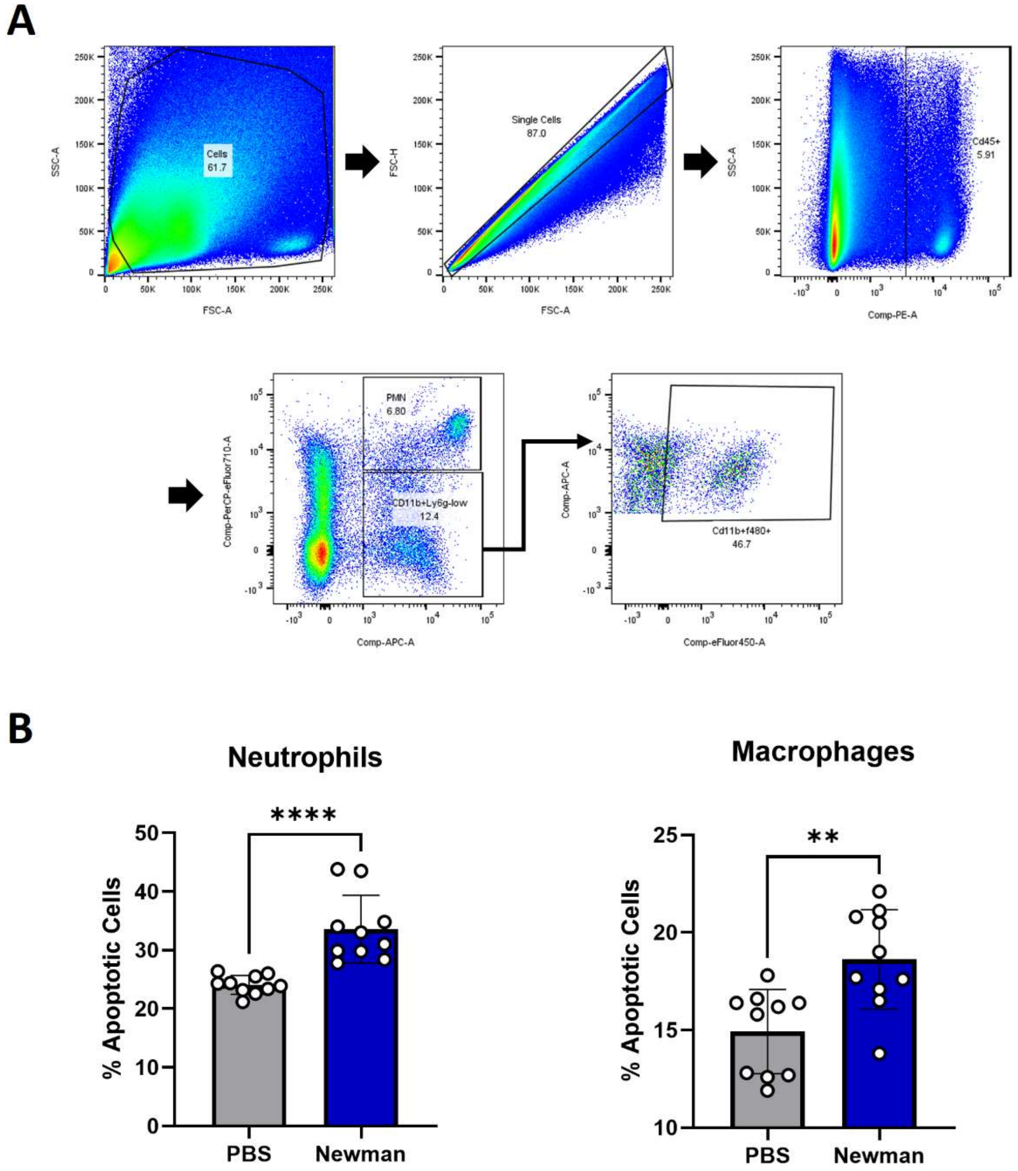

Supplemental Figure 5: NT flow cytometry gating strategy.

Mice were colonised with streptomycin resistant Newman (2 × 10^{8} CFU/nose) and 24 hrs post-colonisation, mice were culled, and NT was excised. Cells isolated from the nasal tissue were stained with extracellular markers with the following gating strategy on Single cells: Cd45+> Cd11b+Ly6g^{hi} or Cd11b+Ly6g$^{Neg\text{-}low}$>Cd11b+F4/80+ (A). % Late apoptotic cells double positive for Apotracker and Zombie NIR viability dye was determined for Cd11b^{+}Ly6g^{hi} neutrophils and Cd11b^{+}F4/80 macrophages (B). Results are expressed as mean ± SD and statistically analysed for animal experiments (10 mice per group) using an unpaired T test for CFU assessment and a Mann-Whitney U test for absolute counts of apoptotic cells (P value ** < 0.01 and **** < 0.0001).

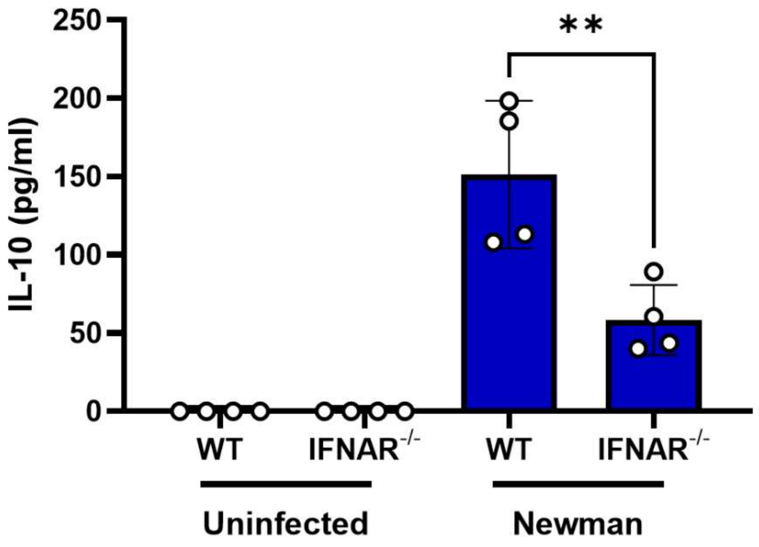

Supplemental Figure 6: *S. aureus* driven IL-10 production in BMDMs in partially IFN-I dependant.

WT and IFNAR-/- BMDMs were infected with *S. aureus* strain Newman at a MOI of 100 for 1 h. BMDMs were then incubated with gentamicin media for 1 h which was then replaced with antibiotic free media. 24 h post gentamicin treatment, cell supernatants were harvested, and IL-10 levels measured by ELISA. Results are expressed as mean ± SD and statistically analysed using a two-way ANOVA with a Šidák multiple comparisons post-test (n=4) (P value ** < 0.01 and **** < 0.0001).

Uncropped Blots

DAXX

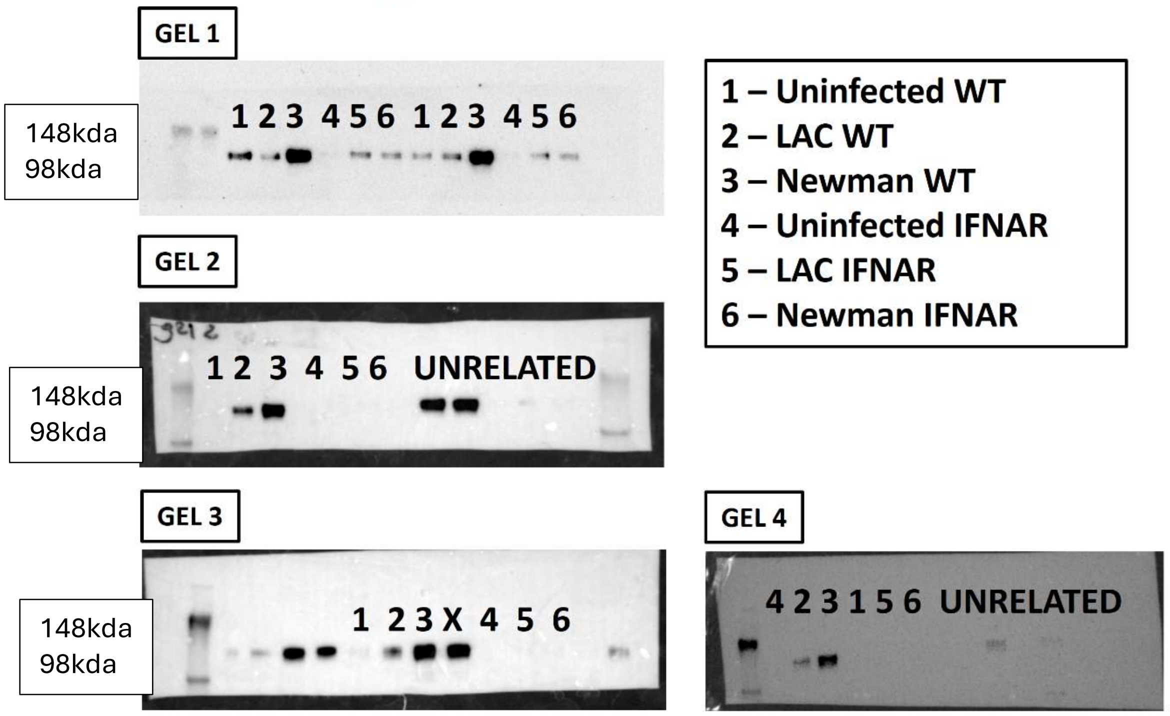

B-ACTIN

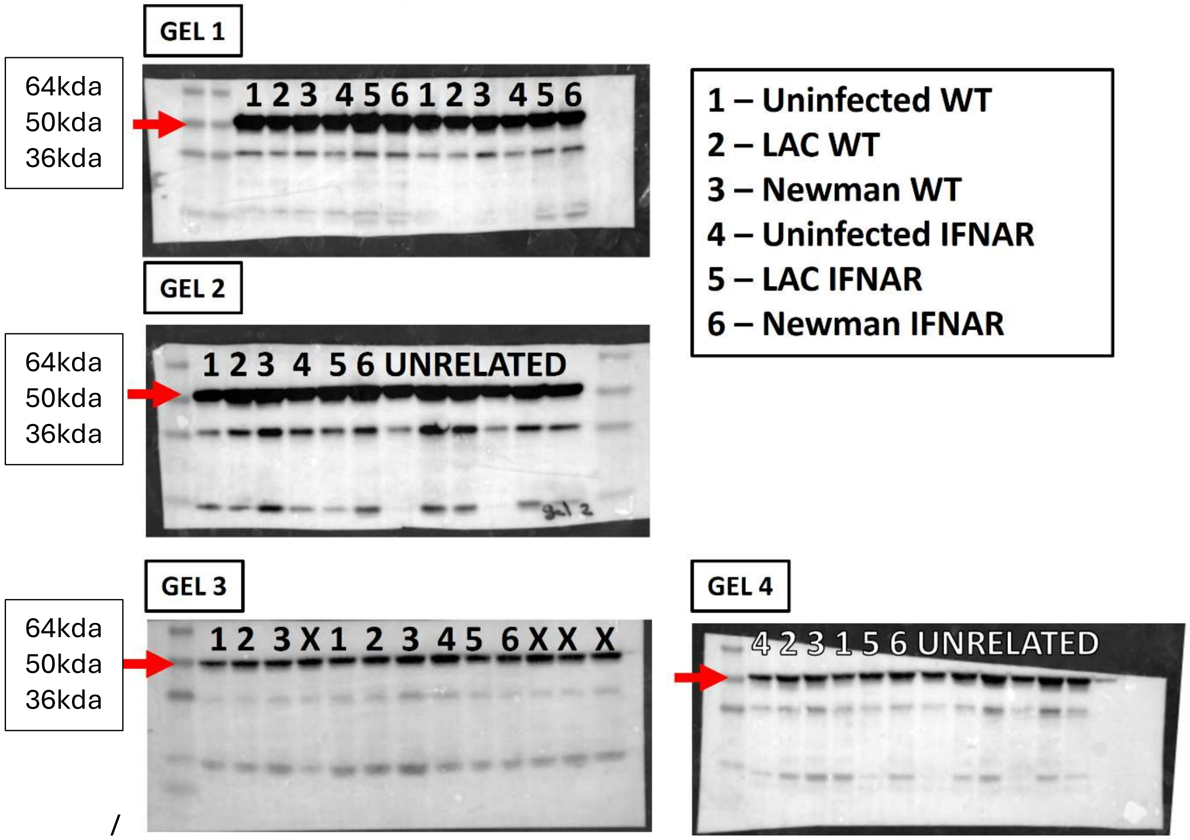

/

CASPASE 3

GEL 2

64kda
50kda
36kda

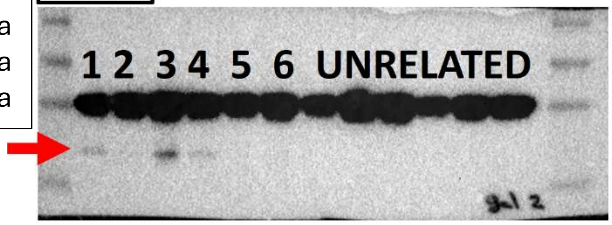

1 – Uninfected WT
2 – LAC WT
3 – Newman WT
4 – Uninfected IFNAR
5 – LAC IFNAR
6 – Newman IFNAR

GEL 3

64kda
50kda
36kda

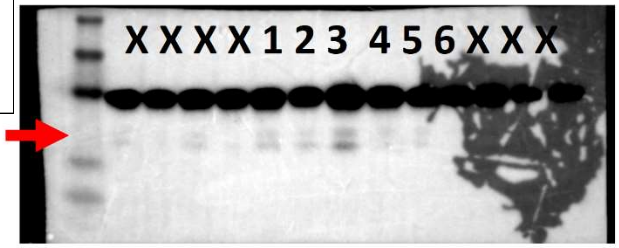

GEL 4

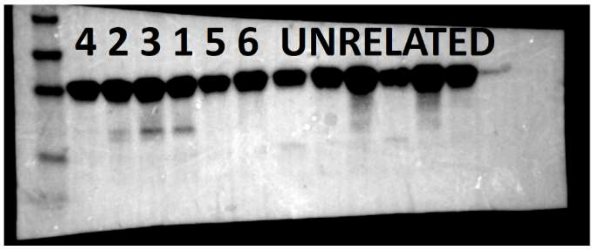

In vivo – Nasal tissue

TRAIL

64kda
50kda
36kda

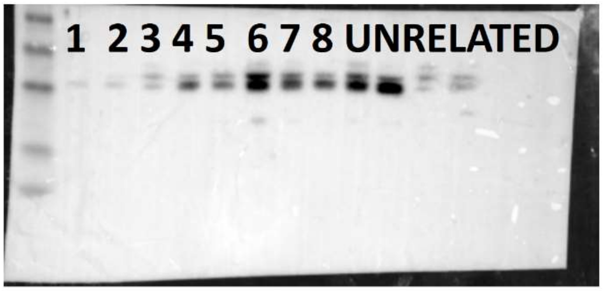

1 – PBS Mouse 1
2 – PBS Mouse 2
3 – PBS Mouse 3
4 – PBS Mouse 4
5 – Newman Mouse 1
6 – Newman Mouse 2
7 – Newman Mouse 3
8 – Newman Mouse 4

Ponceau S total protein

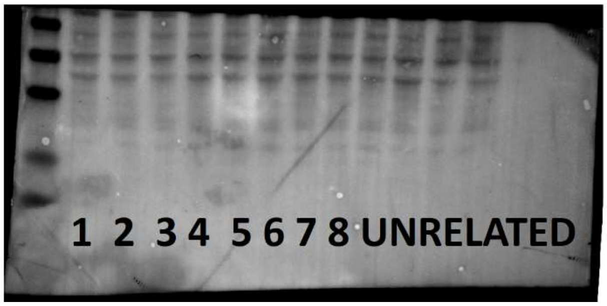

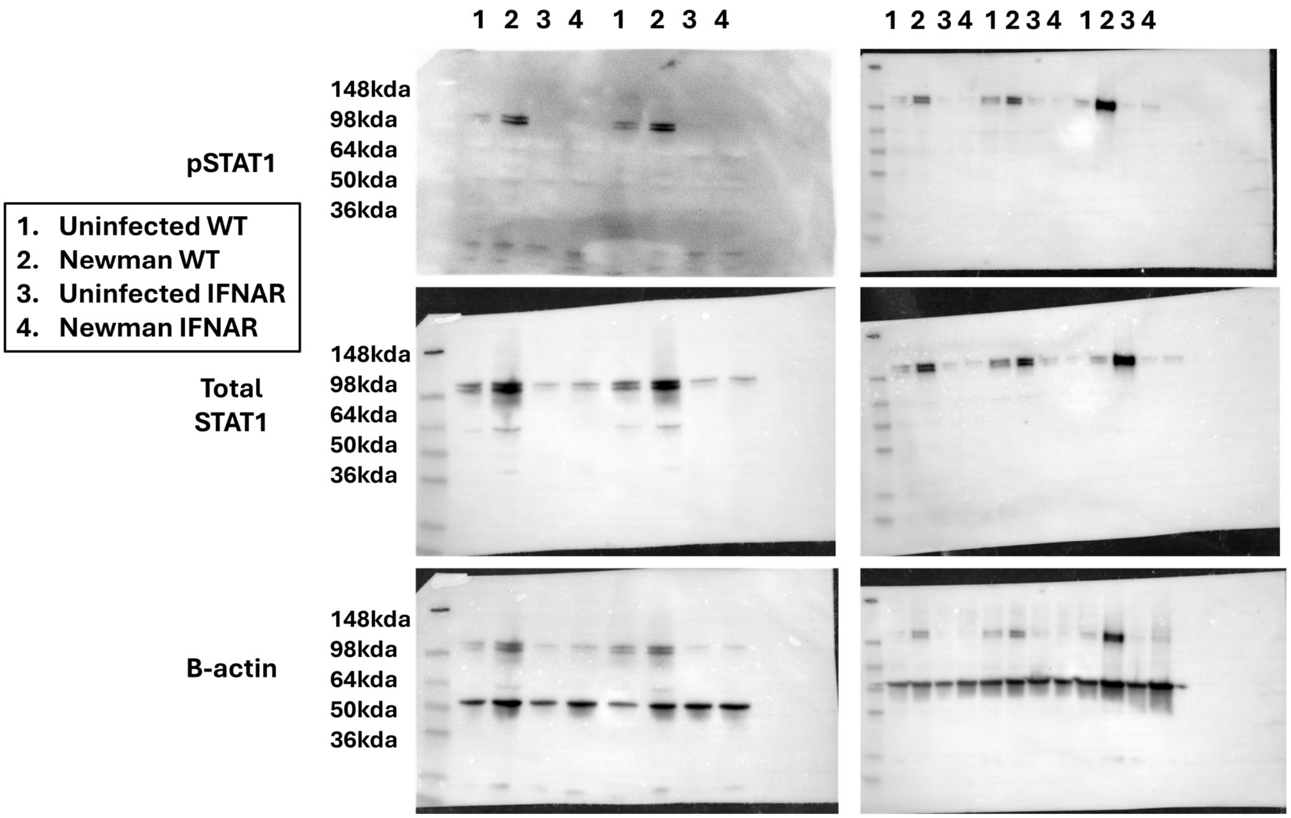
1 2 3 4 1 2 3 4
1 2 3 4 1 2 3 4 1 2 3 4
pSTAT1
Total STAT1
B-actin
148kda
98kda
64kda
50kda
36kda
1. Uninfected WT
2. Newman WT
3. Uninfected IFNAR
4. Newman IFNAR
